# Expression of HTRA Genes and Its Association with Microsatellite Instability and Survival of Patients with Colorectal Cancer

**DOI:** 10.3390/ijms21113947

**Published:** 2020-05-31

**Authors:** Dorota Zurawa-Janicka, Jarek Kobiela, Tomasz Slebioda, Rafal Peksa, Marcin Stanislawowski, Piotr Mieczyslaw Wierzbicki, Tomasz Wenta, Barbara Lipinska, Zbigniew Kmiec, Wojciech Biernat, Andrzej Jacek Lachinski, Zbigniew Sledzinski

**Affiliations:** 1 Department of General and Medical Biochemistry, Faculty of Biology, University of Gdansk, Wita Stwosza 59, 80-308 Gdansk, Poland; tomasz.wenta@biol.ug.edu.pl (T.W.); barbara.lipinska@biol.ug.edu.pl (B.L.); 2 Department of General, Endocrine and Transplant Surgery, Faculty of Medicine, Medical University of Gdansk, Mariana Smoluchowskiego 17, 80-214 Gdansk, Poland; jaroslaw.kobiela@gumed.edu.pl (J.K.); alach@gumed.edu.pl (A.J.L.); zsle@gumed.edu.pl (Z.S.); 3 Department of Histology, Faculty of Medicine, Medical University of Gdansk, Debinki 1, 80-211 Gdansk, Poland; t.slebioda@gumed.edu.pl (T.S.); m.stanislawowski@gumed.edu.pl (M.S.); pwierzb@gumed.edu.pl (P.M.W.); zbigniew.kmiec@gumed.edu.pl (Z.K.); 4 Department of Pathomorphology, Faculty of Medicine, Medical University of Gdansk, Mariana Smoluchowskiego 17, 80-214 Gdansk, Poland; rafalpeksa@gumed.edu.pl (R.P.); wojciech.biernat@gumed.edu.pl (W.B.)

**Keywords:** HtrA serine proteases, colorectal cancer, HtrA3 isoforms, microsatellite instability

## Abstract

HtrA proteases regulate cellular homeostasis and cell death. Their dysfunctions have been correlated with oncogenesis and response to therapeutic treatment. We investigated the relation between HtrA1-3 expression and clinicopathological, and survival data, as well as the microsatellite status of tumors. Sixty-five colorectal cancer patients were included in the study. The expression of *HTRA1-3* was estimated at the mRNA and protein levels by quantitative PCR and immunoblotting. Microsatellite status was determined by high-resolution-melting PCR. We found that the *HTRA1* mRNA level was higher in colorectal cancer tissue as compared to the unchanged mucosa, specifically in primary lesions of metastasizing cancer. The levels of HtrA1 and HtrA2 proteins were reduced in tumor tissue when compared to unchanged mucosa, specifically in primary lesions of metastasizing disease. Moreover, a decrease in *HTRA1* and *HTRA2* transcripts’ levels in cancers with a high level of microsatellite instability compared to microsatellite stable ones has been observed. A low level of HtrA1 or/and HtrA2 in cancer tissue correlated with poorer patient survival. The expression of *HTRA1* and *HTRA2* changes during colorectal carcinogenesis and microsatellite instability may be, at least partially, associated with these changes. The alterations in the *HTRA1/2* genes’ expression are connected with metastatic potential of colorectal cancer and may affect patient survival.

## 1. Introduction

Colorectal carcinoma (CRC) is the third most common cancer and the fourth leading cause of cancer-related deaths worldwide. More than one-million new CRC cases are diagnosed every year, with higher incidence rates in developed western countries. CRC patients may be successfully cured with surgery at early stages; however, CRC is most frequently diagnosed in late stages when prognosis of the disease is poor; the five-year survival rate of metastatic CRC patients (stage IV) does not exceed 15 % [1,2]. Only 5–10% cases of CRC are associated with inherited mutations in known colorectal cancer-related syndromes, but the majority of the patients (approx. 80%) have sporadic forms of the disease, which is thought to be influenced by diet, lifestyle, and environmental factors [2].

CRC evolves through a stepwise accumulation of mutations in multiple genes that are involved in the regulation of critical biological pathways, such as cell proliferation, migration, and apoptosis. There are three genetic pathways that are implicated in CRC pathogenesis: chromosomal instability (CIN), microsatellite instability (MSI), or CpG island methylator phenotype (CIMP) pathway. The CIN tumors are characterized by aneuploidy and loss of heterozygosity, and represent most of sporadic CRC cases. In the MSI pathway, lesions in DNA mismatch repair (MMR) genes lead to an accumulation of mutations in many growth-regulating genes. As microsatellites due to their repetitive nature are prone to errors during DNA replication, inability of the MMR machine to correct these errors is recognized by frameshift mutations in microsatellite repeats [3,4]. The CIMP pathway is associated with promoter hypermethylation of multiple suppressor genes and *v-raf murine sarcoma viral oncogene homolog B1* (*BRAF*) mutation leading to constitutive activation of this kinase [5]. These pathways often overlap in molecular tumor subtypes, which has considerable prognostic implications [6,7]. Since the disease has a heterogenous nature and arises from multiple genetic lesions, the patients’ responses to treatment are diverse and treatment outcome may be far from expectations [2]. Thus, there is an urgent need to better understand molecular basis of colorectal oncogenesis and identify new prognostic markers and therapeutic targets for future individualized treatment strategies.

Human HtrA1-3 proteins belong to the HtrA (High temperature requirement A) family of stress-induced serine proteases widely present in prokaryotic and eukaryotic organisms. Characteristic feature of their architecture is a presence of a chymotrypsin-like protease domain, followed by at least one C-terminal PDZ (Postsynaptic density-95, Disc large tumor suppressor, Zonula occludens-1) domain that is involved in protein-protein interactions. The general function of the HtrAs is to control the quality of proteins; they recognize aberrant proteins and degrade them to avoid the accumulation of toxic aggregates. Apart from that, they process specific substrates and, in this way, regulate many cellular pathways [8]. Human HtrAs are involved in the maintenance of mitochondrial homeostasis, stress response, modulation of extracellular environment, regulation of cell motility, signaling, and death. Disturbances in their functions contribute to the plethora of pathologies, such as neurodegenerative diseases, musculoskeletal disorders, and oncogenesis [9,10,11]. 

Several lines of evidence exist showing that HtrA1 acts as a tumor suppressor. *HTRA1* expression was found to be downregulated or even lost in various primary tumors and metastatic foci [9,12]. In the case of several cancers (e.g., ovarian, gastric and breast tumors), a diminished level of HtrA1 protein in tumor tissue has been correlated with poor response to platinum-based chemotherapy, metastasis, and worse clinical outcome [13,14,15,16,17,18]. Studies on cancer cell lines and animal models showed that a decreased expression of *HTRA1* contributes to the survival of cancer cells, and promotes metastasis, while the upregulation of *HTRA1* facilitates the inhibition of cancer cell proliferation in vitro, tumor growth, and intraperitoneal dissemination [19,20,21,22,23,24]. The association of HtrA1 with carcinogenesis arises from the fact that the protease stimulates the apoptosis of damaged cells [16,21,24], is implicated in modulation of microtubule stability and cell mobility [25], and regulation of cellular signaling mediated by TGF-β proteins [8]—cytokines with a proven role in neoplastic transformation [26].

HtrA2 is a mitochondrial protein that plays a dual role in cell physiology. This protease maintains mitochondrial homeostasis contributing to cell survival; it controls the quality of mitochondrial proteins and affects the biogenesis and dynamics of mitochondria, and its dysfunction was found to be associated with neurodegeneration and age-related diseases [8,10]. On the other hand, HtrA2 might trigger cell death of irreversibly damaged cells by promoting apoptosis. The general mechanism of proapoptotic action of HtrA2 states that, upon action of apoptosis-inducing agents, the protease is released form mitochondria to the cytosol, where it degrades a broad range of the inhibitor of apoptosis proteins (IAPs) and attenuates IAPs-mediated inhibition of caspases facilitating cell death. Additionally, HtrA2 degrades several other antiapoptotic proteins and processes proapoptotic proteins [10]. The expression of *HTRA2* changes during neoplastic transformation; however, it is down-regulated in some tumors and upregulated in others [10,12,27,28,29], which suggests that the *HTRA2* expression varies depending on tumor type. 

HtrA3 is produced by alternative RNA splicing, resulting in two isoforms, a long (HtrA3L) and a short (HtrA3S) one lacking PDZ domain, which makes HtrA3S a unique member of the HtrA family. HtrA3 was previously identified as a pregnancy-associated protease whose dysfunction has been linked with preeclampsia [30,31,32,33]. Disturbances in HtrA3 action were also associated with oncogenesis and this protease is suggested to be a potential tumor suppressor. Similarly to HtrA1 and HtrA2, HtrA3 exhibits proapoptotic function in certain pathological conditions; upon action of chemotherapeutic drugs, the mitochondria-localized HtrA3 is released into the cytosol and it participates in the stimulation of mitochondria-mediated apoptosis of cancer cells [34,35]. The expression of *HTRA3* has been found to be diminished or even lost in several cancer cell lines and tumors [10], which was associated with poor response to chemotherapeutic treatment and higher risk of postoperative recurrence in patients with lung cancer [36,37]. On the other hand, the *HTRA3* expression was found to be significantly up-regulated in other types of tumors [12]. In addition, the *HTRA3* isoforms were found to be differently expressed in ovarian and thyroid tumors [38,39]. Thus, the expression of *HTRA3* might be tumor-type-specific, and this question needs further investigations. Similarly to HtrA1, HtrA3 is also implicated in the regulation of TGF-β signaling and acts as an inhibitor of the pathway [40].

All of the above stress the importance of human HtrAs in oncogenesis. So far, data concerning implication of HtrA proteins in colorectal oncogenesis are limited; very little is known regarding the expression status of the *HTRA1-3* genes in CRC and their relationships with clinicopathological features. In addition, limited knowledge is available on the implication of the HtrA3 isoforms in tumorigenesis, and there is no information regarding their involvement in CRC. Since CRC is one of the most prevalent and deadly cancers worldwide, investigating molecular pathways and proteins associated with the CRC development is of particular interest.

The aim of the study was to elucidate whether the HtrA1, HtrA2, HtrA3L, and HtrA3S proteins are implicated in colorectal oncogenesis by evaluation of expression of the *HTRA1*, *HTRA2*, *HTRA3* genes at the mRNA and protein levels, and the estimation of possible correlations between the expression levels of the tested genes and clinicopathological characteristics of the patients, microsatellite status of a tumor and survival data.

## 2. Results

### 2.1. Expression of the HTRA1 and HTRA2 Genes Is Changed in Colorectal Tumors

We investigated the expression of the *HTRA1*, *HTRA2*, and *HTRA3* genes at both the mRNA and protein levels in CRC tissue and macroscopically unchanged colorectal mucosa from the surgical margin originating from CRC patients. 

**Figure 1:**
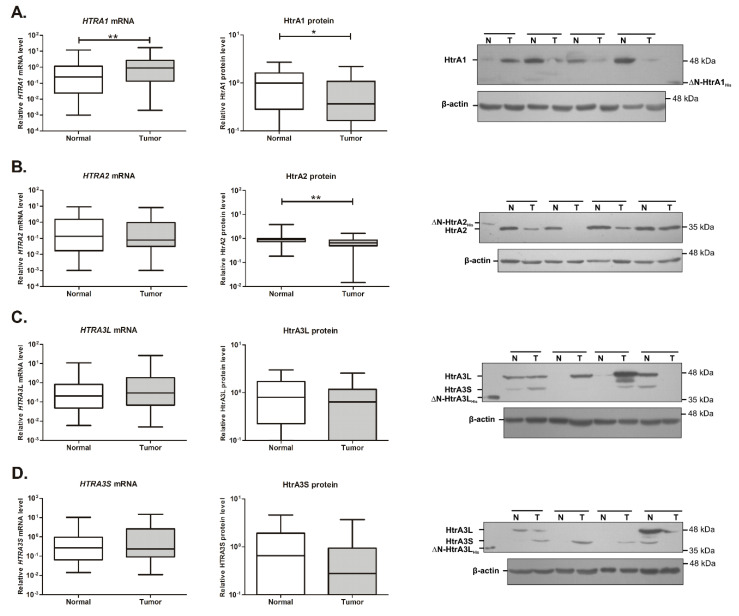
Expression of the *HTRA1* (**A**), *HTRA2* (**B**), and *HTRA3* genes (**C**,**D**) in unchanged colorectal mucosa (Normal) and colorectal carcinoma (CRC) tissue (Tumor). The plot boxes represent the relative mRNA levels of the *HTRA* genes measured using real-time PCR (the left panel of the figure) and the relative levels of HtrA proteins assayed by using western blotting method (the middle panel of the figure). Statistical significance was determined by the Mann-Whitney U test. * *p* < 0.05, ** *p* < 0.01. The right panel of the figure presents examples of detection of HtrA1, HtrA2 and HtrA3 isoforms in unchanged colorectal mucosa (N) and colorectal tumor tissue (T). Tissue lysates containing equal amount of total protein (75 μg) were resolved by SDS-PAGE and subjected to western blotting analysis using antibody against a given HtrA protein or β-actin used as a loading control. To detect HtrA3 an anti-HtrA3 antibody recognizing both HtrA3 isoforms was used. Positions of the purified recombinant ΔN-HtrA proteins (His-tagged HtrA proteins lacking N-terminal domains) used as positive western blotting controls are indicated; ΔN-HtrA1_His_ is indicated on the right side of the blot image whereas ΔN-HtrA2_His_ and ΔN-HtrA3L_His_ are indicated on the left side of the blot images. Positions of the molecular weight standards (kDa) are marked on the right margins of the blots.

The real-time PCR method was used to quantify the relative mRNA levels of *HTRA1*, *HTRA2* and both variants of *HTRA3*: *HTRA3L* and *HTRA3S*. A significant difference in the *HTRA1* mRNA levels between CRC tissue and unchanged colorectal mucosa has been found; the mRNA level of *HTRA1* was 1.8-fold higher in tumor tissue when compared to control tissue (Figure 1A). No significant changes in the mRNA levels of the other *HTRA* genes between tumor and control tissues were found.

We assessed the HtrA proteins’ levels in the CRC tissue and unchanged colorectal mucosa by SDS-PAGE and western blotting. For each tissue sample, a relative protein level of a given HtrA was calculated (for details see the Materials and Methods section). Immunoblotting analysis of the tissue samples with the anti-HtrA1 and anti-HtrA2 antibodies revealed bands of ~50 and ~35 kDa, respectively (Figure 1A,B); the former corresponding to the full-length HtrA1 protein, while the latter to a mature form of the HtrA2 protease comprising the protease and PDZ domains. We used polyclonal anti-HtrA3 antibodies recognizing both HtrA3 isoforms to detect HtrA3 in tissue samples. Analysis of immunoblots revealed bands of ~50 and ~40 kDa that corresponded to the full-length forms of HtrA3L and HtrA3S (Figure 1C,D). These results of immunodetection are in accordance with observations of the previous studies [39,41,42].

The immunoblotting-based analysis revealed that the levels of HtrA1 and HtrA2 proteins were significantly reduced in CRC tissue as compared to unchanged mucosa (Figure 1A,B); specifically, the HtrA1 level was 0.6-fold lower and the HtrA2 level was 0.7-fold lower. Of the analyzed cases, 61 % of colorectal tumors had lower level of HtrA1 when compared to corresponding colorectal mucosa, whereas the HtrA2 protein level was lower in 71 % of the tumors. The levels of the HtrA3 isoforms were slightly reduced in colorectal tumors, but the changes did not reach significance (Figure 1C,D). It should be noted that HtrA3 was not detected in approximately 30 % of tissue samples, both normal and CRC. HtrA1 was not detected in some tissue samples with a higher prevalence in colorectal tumors (specifically, the protein was not detected in approximately 10 % of tumor tissue samples and in 2 % of unchanged colorectal mucosa).

**Figure 2:**
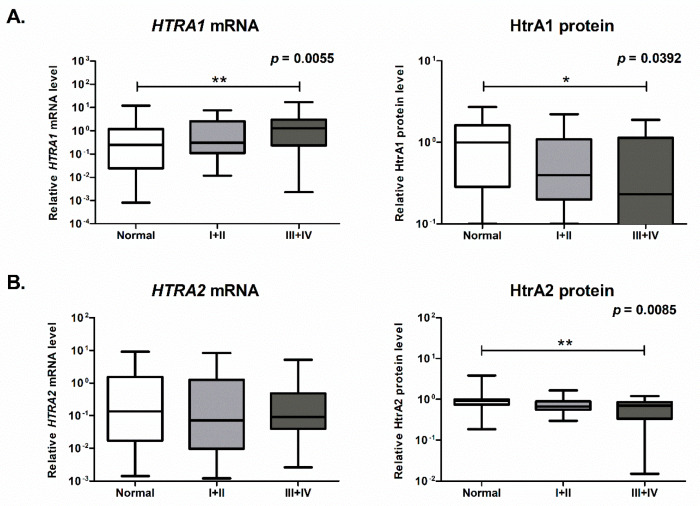
Association of expression of the *HTRA1* (**A**) and *HTRA2* (**B**) genes with metastatic progression of CRC. The plot boxes represent the relative mRNA level of the *HTRA1/2* genes and the relative levels of the HtrA1/2 proteins in unchanged colorectal mucosa (Normal) and tumor tissue of locally advanced lesions (combined stages I and II according to the TNM classification system) and tumor tissue (from primary lesions) derived from patients with metastatic disease (combined stages III and IV). Statistical significance was determined by the Kruskal–Wallis test (the *p*-values are presented in the top right corner of the graphs) and the Dunn’s multiple comparison post hoc test. * *p* < 0.05, ** *p* < 0.01.

Next, we investigated the correlation of the *HTRA* genes with the metastatic potential of CRC. To do this, we analyzed the mRNA and protein levels of HtrAs in tumor tissues from locally advanced tumors (combined stages I and II according to the TNM classification system) and in the tumor tissues of primary lesions from patients with metastatic disease (combined stages III and IV). We found that the *HTRA1* mRNA level was not significantly increased in the locally advanced tumors, but was significantly higher in tumors from patients with metastatic CRC when compared to control mucosa (Figure 2A). Surprisingly, the HtrA1 protein levels in both tumor tissue groups were slightly reduced and they reached significance in primary tumors of metastasizing disease. A significant reduction of the HtrA2 protein level in the primary tumors from patients with metastatic cancer as compared to control non-malignant tissue was observed (Figure 2B). No significant differences in mRNA or protein levels of the *HTRA3L/S* between the CRC and control groups were found.

Thus, these data indicate that the expression of the *HTRA1* and *HTRA2* genes, but not of *HTRA3* undergoes significant alterations during colorectal oncogenesis and suggest that the observed changes might be associated with metastatic potential of CRC.

No significant correlations between the expression of the *HTRA* genes (at both mRNA and protein levels) and patient gender or age have been found. Additionally, we did not find any significant differences in the expression of the analyzed genes between the CRC tissues from the tumors located in the right and left side of the colon.

### 2.2. Location of the HtrA Proteins in CRC Tissue and Colorectal Mucosa

During the course of the study, we also examined HtrA proteins’ location in CRC tissue and unchanged colorectal mucosa by means of an immunohistochemical staining (IHC). The IHC was performed on paraffin-embedded tissues (tumor and unchanged colorectal mucosa from fifteen CRC patients).

**Figure 3:**
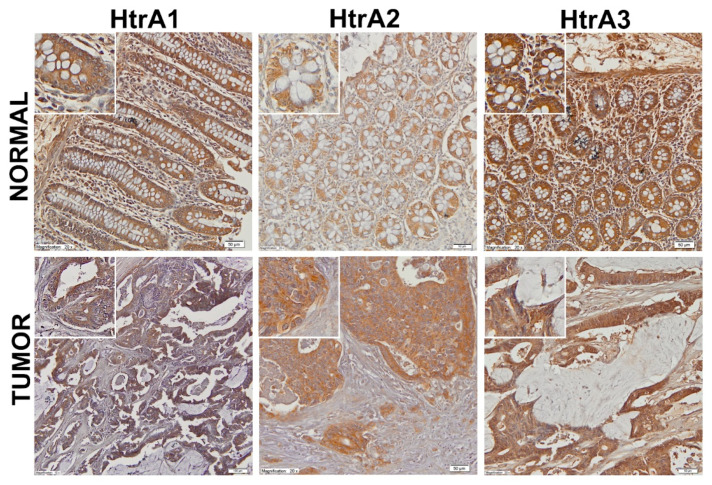
Location of HtrA proteins in normal colorectal mucosa and tumor tissue. HtrA proteins were stained immunohistochemically and, subsequently, tissue sections were subjected to hematoxylin staining according to the protocol included in the Materials and methods section. The matched samples are presented. Original magnification is 200×. Magnification of the insets is 400×. Scale bars = 50 μm.

All of the HtrA proteins showed cytoplasmic immunoreactivity in epithelial compartment of normal colorectal mucosa. Immunoreactive labeling of the HtrA1 and HtrA3 proteins was also observed in stromal compartment (Figure 3). In colorectal tumor tissues, HtrA1 and HtrA3 were detected in both tumor cells and stromal compartment (Figure 3). However, tumor cells and stromal immunostaining intensity was variable among CRC cases. These observations are in agreement with the previously published results [43,44]. In the case of HtrA2, the protein was present in tumor cells, but not detected in stromal compartment, which is compatible with the intracellular location of the protease.

### 2.3. Expression of HTRA1 and HTRA2 Is Associated with Microsatellite Status of the Colorectal Tumor

We checked whether the expression of the *HTRA* genes in colorectal cancers was associated with microsatellite status (MS). The MS status of each CRC case was determined by the polymorphism analysis of the microsatellite loci that was recommended by the National Cancer Institute Workshop and Familial Predispositions [45] (see the Materials and Methods section). Subsequently, we analyzed the mRNA levels of the *HTRA* genes in the microsatellite stable (MSS), low-level microsatellite instability (MSI-L), and high-level microsatellite instability (MSI-H) cases. 

**Figure 4:**
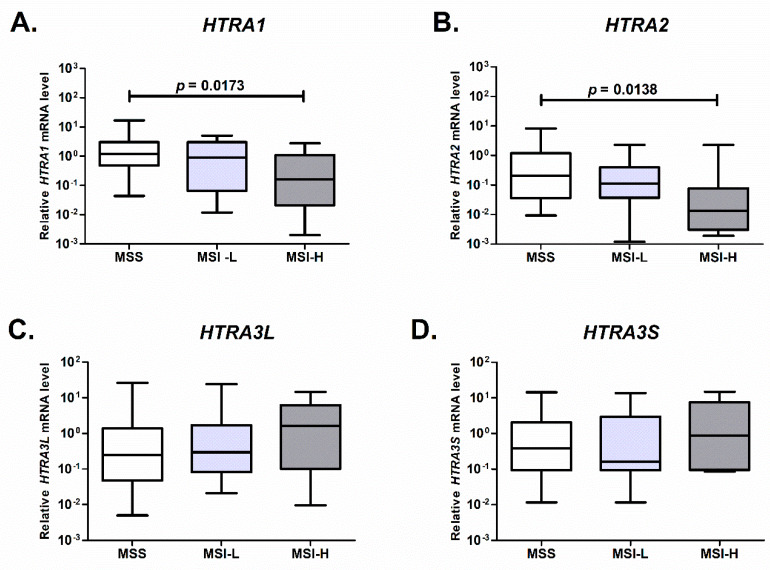
Association between expression of the *HTRA1* (**A**), *HTRA2* (**B**), and *HTRA3* genes (**C**,**D**) genes and microsatellite status of colorectal tumors. The plot boxes present the relative mRNA levels of the *HTRA* genes in MSS (*n* = 31), MSI-L (*n* = 23) and MSI-H (*n* = 17) colorectal cancers. Statistical significance was determined by the Mann-Whitney U test.

We found that the mRNA level of *HTRA1* was lower in the MSI-H tumors as compared to the MSS ones (Figure 4A). Since the overall expression of *HTRA1* was found to be increased in tumor tissue (Figure 1A), we compared mRNA level of the gene in control mucosa with the mRNA levels in the MSS and MSI tumors, and found that the *HTRA1* transcript level was significantly higher in the MSS tumors than in the control tissue (*p* < 0.01). 

In case of *HTRA2*, the mRNA level of the gene was decreasing with an increased microsatellite instability status of CRC. The level of *HTRA2* transcript was diminished in the MSI-H group when compared to the MSS ones (Figure 4) We did not find any significant differences in the mRNA levels of *HTRA3L/S* between the analyzed groups.

These results indicate that in colorectal carcinogenesis microsatellite status of a tumor is connected with the expression of the *HTRA1* and *HTRA2* genes and suggest that the microsatellite instability pathway might be involved in regulation of these genes in CRC.

### 2.4. Survival of CRC Patients Correlates with the Levels of HtrA1 and HtrA2 Proteins

**Figure 5:**
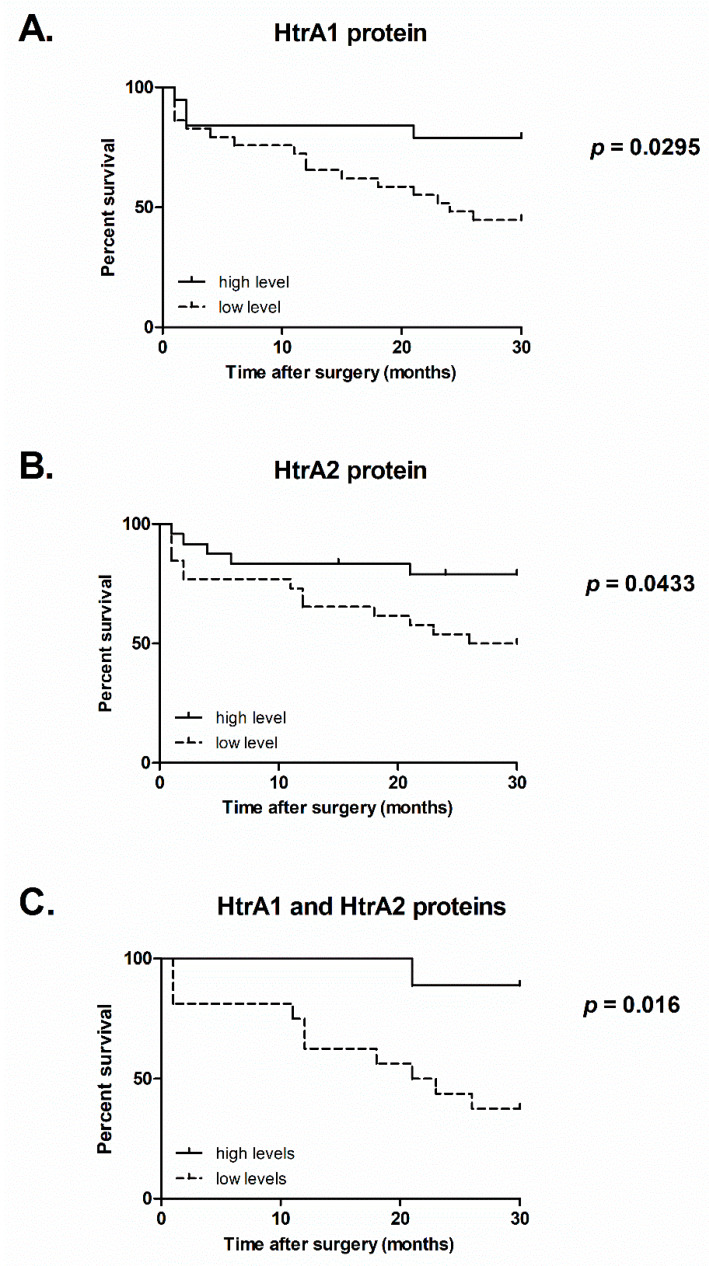
Analysis of the CRC patients’ survival in relation to protein levels of HtrA1 (*n* = 48) (**A**), HtrA2 (*n* = 50) (**B**), and levels of both, HtrA1 and HtrA2 proteins (*n* = 25) (**C**). Statistical significance was evaluated using log-rank Mantel-Cox test and p values are presented. The discrepancy between numbers of CRC patients, included in the survival analysis in relation to HtrA1 level (*n* = 50) and HtrA2 level (*n* = 48) is due to the fact that some tumor tissue lysates degraded markedly and were not processed for the immunoblotting-based analysis of the HtrA2 protein. Analysis of survival in relation to levels of both proteins simultaneously were performed on a group of 25 individuals who had low levels of both HtrA1 and HtrA2 proteins (*n* = 16) or high levels of the HtrA proteins (*n* = 9). The CRC patients with low level of HtrA1 and, simultaneously, high level of HtrA2, or vice versa were not included in the analysis.

We checked whether the expression of the *HTRA* genes had an impact on the survival of the CRC patients. We categorized the CRC cases into two groups: tumors with “low” and tumors with “high” expression of a given *HTRA*, depending on the mean value of mRNA or protein level, and monitored survival of the patients within 30 months. The survival analysis showed that the CRC patients with low level of the HtrA1 protein (protein level lower or equal to 0.6-fold decrease as compared to control group) had a significantly lower survival rate (45%) than the patients with a high HtrA1 level (79%) (Figure 5A). Additionally, the patients with a decreased HtrA2 protein level (protein level lower or equal to 0.7-fold decrease when compared to control group) had a significantly worse survival rate (50%) than the patients with high level of HtrA2 (79 %) (Figure 5B). We also found that the patients with low levels of both HtrA1 and HtrA2 proteins (n = 16) had worse survival (38%) compared to those with high levels of the HtrA proteins (n = 9) (89%) (Figure 5C). We found no significant correlations between the levels of the *HTRA1* and *HTRA2* transcripts and patient survival. Additionally, no differences in the CRC patient survival regarding *HTRA3L/S* genes’ expression (both at the mRNA and protein levels) were found.

## 3. Discussion

In the present study we evaluated the expression of the *HTRA1-3* genes, encoding human HtrA serine proteases HtrA1, HtrA2, and HtrA3 isoforms (HtrA3L and HtrA3S), at both mRNA and protein levels, in colorectal tumor tissue and unchanged colorectal mucosa of the CRC patients. Subsequently, the expression of *HTRAs* was analyzed in relation to clinicopathological parameters and the survival of the patients. Additionally, we analyzed the relationship between the *HTRA* genes’ expression and microsatellite status of CRCs.

We found that the *HTRA1* transcript level was significantly higher in the colorectal tumor tissue than in the unchanged colorectal mucosa (Figure 1A), specifically in tumor tissue from patients with metastatic CRC, with metastases present in lymph nodes and distant organs (combined stages III and IV) (Figure 2A). These results suggest that transcription of the *HTRA1* gene is correlated with colorectal oncogenesis and may be associated with metastatic progression of the disease. The results of numerous studies clearly demonstrated that HtrA1 might function as a stress-induced protease and proapoptotic factor. *HTRA1* gene expression has been found to be elevated in response to stressful conditions induced by a number of agents, for example, upon treatment with anticancer drugs (i.e., cisplatin, doxorubicin, paclitaxel) [16,46], in response to estrogen-induced acute oxidative stress [47], and upon hypoxia-induced stress [48]. The observed elevation in the *HTRA1* transcript level in CRC tissue seems to be consistent with these studies. It can be hypothesized that the increased transcription of the *HTRA1* gene might result from the induction of the pathways activated in response to geno- and proteotoxic stresses accompanying colorectal oncogenesis to counteract cellular damages. It is also in agreement with the general protective role of HtrAs in stressful conditions. 

Somewhat surprisingly, the overall HtrA1 protein level was significantly lower in CRC tissue as compared to the control (Figure 1A), specifically in primary tumors of metastatic disease (Figure 2A). Moreover, a reduced level of HtrA1 in colorectal tumors was found to be correlated with worse patient survival (Figure 5A). Although this result is intriguing, it could be explained by the fact that, in non-pathological conditions, the mRNA level might not correlate with the level of corresponding protein due to multiple mechanisms controlling post-transcriptional processes and degradation events [49]. Such discrepancy has been observed for many genes in cancer cells and tumors, and other diseases as well [50,51,52]. Additionally, in tumor cells, biological noise increases upon tumorigenesis. It facilitates the adaptation of cancer cells to cellular stresses accompanying neoplastic changes (e.g., genetic abbreviations, proteotoxic, and oxidative stresses) and environmental fluctuations that are caused by tumor-infiltrated stromal and immune cells, and contributes to cancer cells’ survival due to the activation of pathways that favor survival instead of apoptotic death [53,54,55]. In this context, the observed reduced level of HtrA1, a protein with a proapoptotic function, might be an element of cancer cell adaptation to avoid death during colorectal oncogenesis. Unfortunately, molecular factors that impair pathways spanning the *HTRA1* mRNA production to the protease synthesis and/or degradation are unknown and remain to be determined.

Our results showing a decrease in HtrA1 protein level in colorectal tumors are in agreement with results of several groups and our previous findings demonstrating a reduction of the protease content or even loss in other primary tumors [9]. Our findings are also consistent with recent results of [43], who analyzed the HtrA1 protein content in CRCs and colorectal mucosa by using IHC staining of tissue slides. Their results revealed that the HtrA1 protein level was lower in colorectal tumors. They also found that HtrA1 localizes in both epithelial and stromal compartments of colorectal mucosa and tumor tissue. The fact that HtrA1 resides intra- and extracellularly emphasizes its importance during colorectal oncogenesis. Recently, Schmidt et al [56] demonstrated that the down-regulation of HtrA1 drives polyploidy and correlates with centrosome amplification in colorectal cancer cells, which makes a direct connection between HtrA1 deficiency and elevated genetic instability in colorectal oncogenesis. Since HtrA1 undergoes secretion, its role in extracellular space can not be ignored. Our IHC results confirmed HtrA1 presence in the extracellular space of tumors and normal mucosa (Figure 3). HtrA1 generally acts as an inhibitor of TGF-β signaling, which has a suppressive role at early steps of colorectal transformation, but promotes tumor progression at late stages [57,58]. Taking into consideration the above facts, HtrA1 might be implicated in colorectal oncogenesis at multiple stages of tumor development and generally act as an anticancer factor. Thus, the observed reduced HtrA1 protein level in colorectal tumors might promote cancer development and progression. Our finding that the CRC patients with lower HtrA1 protein level had a shorter survival supports this hypothesis.

Our results confirmed the intracellular location of HtrA2 (Figure 3) and showed that, in CRC tissue, the HtrA2 protein level was significantly decreased when compared to the unchanged colorectal mucosa (Figure 1B), especially in tumors from patients with metastatic disease (combined stages III and IV) (Figure 2B). In addition, the CRC patients with a lower level of HtrA2 had a shorter survival (Figure 5B). These results suggest the importance of HtrA2 protease in colorectal oncogenesis and an association of the protease with metastatic potential of the cancer and patient outcome.

The implication of HtrA2 in oncogenesis results from its involvement in mechanisms of cell death. HtrA2 participates in apoptosis at multiple stages of intrinsic and extrinsic pathways due to its serine protease activity, while being sequestered in mitochondria and after its release into the cytosol as well, by the degradation of antiapoptotic proteins and activation of proapoptotic proteins [8,10]. The best characterized mechanism of proapoptotic action of HtrA2 relies on mitochondrial release of the protease into the cytosol and degradation of IAP proteins and subsequent activation of caspases. The overexpression of IAPs was found in majority of cancers and connected with poor prognosis. An enhanced expression of a number of IAPs, including cIAP1, IAP2, survivin, XIAP in colorectal tumors has been correlated with worse survival, disease recurrence, and liver metastasis, which defines poorer prognosis [59]. All of the IAP proteins mentioned have been demonstrated as substrates for HtrA2 [10]. In addition, HtrA2 has been identified as a regulator of anoikis that is involved in oncogenic *KRAS*-driven malignant transformation of intestinal epithelial cells (IECs) [60,61]. The detachment of IECs induces mitochondrial release of HtrA2 along with other mitochondrial proteins, such as cytochrome c, and the subsequent promotion of anoikis [60]. Thus, the protease seems to be an important player that attenuates the metastatic potential of cancer cells, and the observed reduction in HtrA2 protein level in colorectal tumors could promote cancer development and dissemination. Thus, HtrA2 and its proteolytic activity appears as a potential target for developing new CRC treatment strategies.

In this study, we also analyzed the expression of the *HTRA* genes in relation to microsatellite status of the CRC, because higher levels of microsatellite instability are associated with increased susceptibility to additional mutations and may affect the expression levels of different genes [62,63]. We found that the mRNA level of the *HTRA1* and *HTRA2* genes significantly decreased with the loss of microsatellite stability (Figure 4A,B). As the microsatellite instability reflects impairment of the DNA repair system and accumulation of mutations, leading to CRC development, the observed decrease of the *HTRA1/2* transcription could be an effect of these mutations. Since protein products of the *HTRA* genes are implicated in mechanisms of stress response and cell death, the attenuation of their expression might be favorable for the development and progression of CRC.

We also analyzed the expression of the *HTRA3* gene in colorectal tumors. The analysis concerned both HtrA3 isoforms, the HtrA3L and HtrA3S. The majority of previously published studies concerning the expression of *HTRA3* in tumor tissue did not focus on the HtrA3 isoforms, but analyzed overall expression, including recently published results based on IHC analysis of HtrA3 content in colorectal tissue. These results highlighted striking differences in HtrA3 content in epithelial and stromal compartments between tumor core, invasive front, and tumor budding area [44,64], and revealed that a higher stromal HtrA3 presence in tumor core was correlated with a high-grade tumor budding and poorer outcome of patients with II stage CRC [64]. In the present study, we confirmed by IHC staining that HtrA3 was present in stroma of tumors (Figure 3) but did not find any significant differences in the level of the HtrA3L/S isoforms between tumor tissue and control mucosa either at mRNA or protein levels. However, we used western blotting technique to estimate the levels of the long and short isoforms and this method did not show the histological location of these isoforms. Unfortunately, specific antibodies differentiating the HtrA3L and HtrA3S are not available yet. Nevertheless, we believe that our results complement sparse data regarding the implication of HtrA3 in colorectal oncogenesis.

In conclusion, we demonstrated that during colorectal oncogenesis expression of the *HTRA1* and *HTRA2* genes undergoes significant changes. The expression of the *HTRA1/2* genes diminished as the microsatellite instability of tumor advanced. Most importantly, the HtrA1/2 protein levels were decreased in tumors; specifically, a significant decrease occurred in the advanced cancer stage (III and IV). Furthermore, the decrease of the HtrA1/2 protein levels had a negative (and cumulative) effect on the survival time of the CRC patients. No significant changes in the levels of the HtrA3L and HtrA3S transcripts or proteins in CRC as compared to controls were observed. These findings indicate that the HtrA1 and HtrA2 proteins may be involved in CRC and they are consistent with earlier studies showing pro-apoptotic and anti-cancer function of these proteins. We believe that these results augment our knowledge concerning the implication of the HtrA proteins in colorectal oncogenesis and might serve as the starting point for further studies to confirm their usefulness as potential prognostic markers.

## 4. Materials and Methods 

### 4.1. Patients and Specimens

The CRC tissue samples and macroscopically unchanged mucosa from the surgical margin were collected from CRC patients (*n* = 65) surgically treated at the Department of General, Endocrine, and Transplant Surgery of the Medical University of Gdansk, Poland. Tumors that were located in the anal canal and anus were not included in the study. The patients were not treated with neoadjuvant chemo- or radiotherapy. One part of every tissue sample was fixed in formalin and used for histopathological and immunohistochemical examination; the second part was immersed in RNA-stabilizing solution (RNA Later Solution, Life Technologies, Warsaw, Poland), stored at −70 °C for further mRNA and protein assays. The tissue samples underwent standard histopathological evaluation at the Department of Pathomorphology of the Medical University of Gdansk and tumor staging was defined according to the TNM classification system [65]. Table 1 presents clinical characteristics of the CRC patients. The study was approved by the Independent Bioethics Committee for Scientific Research at the Medical University of Gdansk, Poland (Approval no. NKBBN/239/2015, 26-May-2015).

### 4.2. RNA Isolation and cDNA Synthesis

Total RNA was extracted from tissue samples while using the Total RNA Purification Kit (A&A Biotechnology, Gdynia, Poland). The RNA samples were treated with DNase I as described in [42], total RNA was spectrophotometrically quantified at 260 nm (Epoch spectrophotometer, BioTek, Swindon, UK), and its quality was verified electrophoretically in agarose gels [66]. The total RNA was reverse-transcribed using the Revert Aid First Strand cDNA Synthesis Kit according to the supplier’s protocol (ThermoScientific, Warsaw, Poland). Briefly, 1 μg of total RNA was reverse-transcribed using 200 U of M-MuLV reverse transcriptase in a presence of 0.5 mM oligo(dT)_18_ primer (Sigma–Aldrich, Poznan, Poland).

### 4.3. Real-Time PCR

The quantification of mRNA levels of the *HTRA* genes was performed by SYBR Green-based chemistry and StepOne Plus Real-Time PCR Systems (Life Technologies-Applied Biosystems, Carlsbad, CA, USA). PCR reactions were performed using SensiFAST SYBR No-Rox Kit (BioLine, Nottinghan, UK), according to the manufacturer’s recommendation, and Table 2 presents the primer pairs. The reaction mixture (20 μL) included 1 μL of undiluted cDNA, 0.195 μM concentration of each forward and reverse primer and 10 μL of real time master mix. The amplification parameters included: initial denaturation at 95 °C for 3 min., followed by 40 cycles of denaturation at 94 °C for 10 s, annealing at temperature specific for a given pair of primers (Table 2) for 15 s, extension at 72 °C for 30 s. The amplification of unspecific products was excluded by means of melt curve analysis. Dynamic melt curve analysis was performed for all reactions. All of the reactions were performed in triplicate. The data were automatically collected and analyzed with StepOne Software v.2.2.2 (Applied Biosystems, Warsaw, Poland). The Ct values of the analyzed genes were normalized to the geometric mean of Ct values of *IPO8* and *PPIA* genes, which showed the highest stability in the tested biological samples among other evaluated housekeeping genes [67]. Subsequently, 2^−ΔCt^ values of an individual patient in tumor tissue group were normalized to the average 2^−ΔCt^ value in control group.

### 4.4. Preparation of Tissue Extracts

The protein extracts were prepared using a modified protocol described previously by [69]. Briefly, approximately 30 mg of tissue was washed with GTE buffer containing 50 mM Tris/HCl pH 7.4, 10% glycerol (*v/v*) and 1 mM EDTA, and subsequently immersed in LB buffer containing 50 mM Tris/HCl pH 7.4, 7 M urea, 1% dithiothreitol, 0.8% citric acid, and protease inhibitor cocktail (Roche, Warsaw, Poland). The tissue was homogenized with glass beads and centrifuged (15000× *g*, 30 min.) at 4 °C. Supernatant was collected, stored at −70 °C, and then subjected to protein analysis.

### 4.5. Protein Assay and Electrophoresis

The proteins were quantified by Amido Black staining, and SDS-PAGE electrophoresis was carried out according to Laemmli using standard protocols [66]. Molecular weight standards that were purchased from Blirt S.A. (Gdansk, Poland) were used.

### 4.6. Western Blotting

Immunoblotting analysis of protein extracts was performed, as described previously [39,41,42]. Briefly, samples of tissue lysates containing equal amounts of total protein were resolved by SDS-PAGE and electrotransferred onto Immobilon membrane. Samples of tumor and normal tissues were resolved on the same gel, together with a reference sample. The same sample was used as the reference in all assays for a given HtrA protein. The chosen reference sample was the normal tissue, in which the analyzed protein’s level was close to average. Proteins that were bound to the membrane surface were stained by a standard procedure with the Ponceau S dye in order to confirm proper sample loading, protein resolution by SDS-PAGE and electrotransfer, and also to check whether the protein amounts in the samples were comparable. Polyclonal rabbit immunoglobulins against a given HtrA protein were used as primary antibodies. Anti-HtrA1 immunoglobulins were purchased from Abcam (Burlingame, CA, USA), and antibodies against HtrA2 and HtrA3 were raised by us, as described previously [42,70]. Goat anti-rabbit HRP-conjugated IgG (Sigma-Aldrich, Poznan, Poland) were used as the secondary antibodies. To visualize antigens chemiluminescence detection was performed while using Lumi-Light Western Blotting Substrate (Roche, Warsaw, Poland). The blots were also probed with polyclonal antibody against actin used as a loading control. Rabbit anti-actin HRP-conjugated antibody was purchased from Sigma–Aldrich (Poznan, Poland). The relative levels of the HtrA proteins were calculated as a ratio of the tested protein band intensity to the intensity of a corresponding HtrA protein band in the reference sample resolved on the same gel, and then the values of an individual patients in tumor tissue group were normalized to the average value in control group. The protein fold change value was estimated to distinguish between tumors with low and high expression of a given HtrA. To do this, the relative protein level values of individual patients in colorectal cancer group were used to calculate an average value. The relative protein level that was lower or equal to the calculated mean value was considered low and the relative protein level higher than the mean value was considered high.

### 4.7. Immunohistochemical Staining

Formalin-fixed, paraffin-embedded tumor tissue samples, and unchanged colorectal mucosa from fifteen CRC patients were cut into 4 µm-thick slides, deparaffinized and subjected to immunohistochemical (IHC) staining, as described previously [71]. Briefly, the sections were deparaffinized in xylene and dehydrated in graded alcohol. Antigens were retrieved by heating the sections in 0.01 M citrate buffer (pH 6.0). Endogenous peroxidase activity was quenched with 3% hydrogen peroxide for 10 min. Next, sections were blocked by 2.5% goat serum solution (Vector Laboratories, Burlingame, CA, USA) for 30 min. and then incubated with rabbit polyclonal immunoglobulins against HtrA1, HtrA2, or HtrA3 at concentration of 1:200 for 60 min. Subsequently, sections were incubated with horseradish peroxidase (HRP)-conjugated secondary antibody (Dako, Carpinteria, CA, USA) at concentration 1:100 for 30 min. To visualize antigens DAB (3,3′–diaminobenzidine) HRP Substrate Kit (Vector Laboratories, USA) was used followed by hematoxylin staining.

### 4.8. Microsatellite Instability Typing by High-Resolution-Melting PCR

The determination of the MSI status was based on polymorphism analysis of five microsatellite markers, including BAT26 and D2S123 for *MSH2*, BAT25 for the *c-kit* oncogene, APC-D5S346 for *APC*, and MFd15-D17S250 for *BRCA1*, as recommended by the National Cancer Institute Workshop and Familial Predispositions [45]. MSI status was assessed while using the high-resolution-melting qPCR method, as described by [72] with some modifications, and using recommended PCR oligonucleotides [73]. The total DNA was extracted from paired tumor and normal resection specimens using Total DNA Purification Kit (A&A Biotechnology, Gdynia, Poland). PCR reaction contained 200 nmol/L of each primer and 15 ng of total DNA as a template in a final volume of 10 μL, and it was performed using SensiFast HRM Kit, according to the manufacturer’s recommendations (BioLine, Nottinghan, UK). All of the reactions were carried out in qPCR 8-tube stripes (4titute, UK), and run in duplicate. Firstly, the PCR reactions were run in MyGo Pro Real-Time PCR System (IT-IS International Ltd., UK) with the following time-temperature conditions: 95 °C for three minutes, 35 cycles of 95 °C for 10 seconds, 51 °C for 10 s, and 72 °C for 15 (fluorescence reading). HRM profile: from 60 °C to 97 °C with fluorescence reading every 0.05 °C. The MSI pattern was assessed by auto high resolution melt with the use of MyGoPro PCR Software ver. 3.3 (IT-IS International). Furthermore, HRM analysis was duplicated (by transferring the PCR stripes with PCR products) at StepOne Plus PCR System (Life Technologies-Applied Biosystems, Carlsbad, CA, USA) with the following profile: from 60 °C to 95 °C with fluorescence reading every 0.2 °C, and HRM analysis was assessed by High Resolution Melting software ver. 3.0 (Life Technologies-Applied Biosystems) while using manual of auto alignment of melt curves. The occurrence of MSI was noted if an additional melting spike appeared in tumor samples. The PCR reaction was repeated if the results of the analyses at MyGo and StepOne Plus PCR Systems were different. Differences in the DNA pattern were evaluated as MSI and categorized as low MSI (MSI-L; one or two markers affected) and high MSI (MSI-H; three or more markers affected). If none of the markers was affected, the tumor was acknowledged as microsatellite stable (MSS).

### 4.9. Statistical Analysis

The data were tested for normality using a Kolgomorov–Smirnov test. The Kruskal–Wallis test was used to determine the statistical significance of difference in measured variables between groups followed by Dunn’s multiple comparison test. Differences between two groups were analyzed using Mann–Whitney U test. The postoperative survival rate was analyzed using the Kaplan–Meier method and differences in the survival curves were assesses with the log-rank test according to a Mantel–Cox model. Difference was considered to be significant at *p* < 0.05. The statistical analysis was performed using GraphPad Prism 5 software package (GraphPad Software Inc., San Diego, USA).

## Figures and Tables

**Table 1:** Characteristics of the patients.

Patients Demographics	No. of Cases - *n* (%)
Total	65 (100)
Gender	
Male	41 (63)
Female	24 (37)
Age	
Years ± SD	67 ± 10.5
Tumor stage *	
I + II	30 (46)
III + IV	35 (54)
Anatomical location	
Right-sided	29 (45)
Left-sided	36 (55)

* Tumor staging was defined according to the TNM classification system [65].

**Table 2:** The primer sequences and reaction conditions for real-time PCR.

Gene	Oligonucleotide Sequence 5′→ 3′	Annealing Temp. (°C)	PCR Product Size (bp)	RefSeq	Ref.
*HTRA1*	*Forward:* CGGAAGATGGACTGATCGTGAC *Reverse:* GGTGATGGCTTTTCCTTTGGCC	62	506	NM_002775.4	[42]
*HTRA2*	*Forward:* CCCTATCTCGAACGGCTCAGG *Reverse:* CCATGCTGAACATCGGGAAAGC	63	639	NM_013247.4	[42]
*HTRA3L*	*Forward:* AGATCAAAGACTGGAAGAAGCG *Reverse:* ATGATGTCACCATCTTGGATGC	60	181	NM_053044.1	This work
*HTRA3S*	*Forward:* ATCCATCCCAAGAAAAAGCTCC *Reverse:* CTCAATGAACTGCCAGTGAGG	60	130	NM_001297559.1	This work
*PPIA*	*Forward:* CTTGGGCCGCGTCTCCTTTGAG *Reverse:* GCTTGCCATCCAACCACTCAGTC	59	329	NM_001300981.1	[68]
*IPO8*	*Forward:* TTGGAAGAAACCGCGCTTGAGG *Reverse:* ACCAGGCTGCATCTCGACTCTG	59	118	NM_001190995	[68]
